# Supplementary material for: Critique of enhanced power claimed for Quasi-ANCOVA and Dual-Centered ANCOVA
Source: PLoS One. 2025 Jan 22;20(1):e0317860. doi: 10.1371/journal.pone.0317860 (PMC11753683; doi:10.1371/journal.pone.0317860)
Supplement: S1 File — (DOCX) [file pone.0317860.s001.docx]

########## Monte Carlo simulation to determine type I errors

########## Author: Hua Lin

library(tidyverse)

## set up parameters

N <- 1000 #repeat

NT <- 1000 # total sample size

NG <- 500 # Sample size for girls

NB <- NT-NG # sample size for boys

M0g <- 130 # Pretest mean for girls

M1g <- 130 #post test mean for girls

M0b <- 130 # Pretest mean for boys

M1b <- 130 #post test mean for boys

SD <- 15 # standard deviation

output <- matrix(nrow = N,ncol = 45)

colnames (output)<- c("rho",

"an_b_girl", "an_s_b_girl", "an_TSS", "an_SSREG_girl", "an_SSE", "an_MSE", "an_v_error",

"ac_b_y0", "ac_s_b_y0", "ac_b_girl", "ac_s_b_girl", "ac_TSS", "ac_SSREG_y0", "ac_SSREG_girl", "ac_SSE", "ac_MSE", "ac_v_error",

"d_b_girl", "d_s_b_girl", "d_TSS", "d_SSREG_girl", "d_SSE", "d_MSE", "d_v_error",

"q_b_y0", "q_s_b_y0", "q_b_girl", "q_s_b_girl", "q_TSS", "q_SSREG_y0", "q_SSREG_girl", "q_SSE", "q_MSE", "q_v_error",

"c_b_y0", "c_s_b_y0", "c_b_girl", "c_s_b_girl", "c_TSS", "c_SSREG_y0", "c_SSREG_girl", "c_SSE", "c_MSE", "c_v_error")

smry <- matrix(nrow = 19 ,ncol = 45)

tsd <- matrix(nrow = 19 ,ncol = 5)

d <- 1

id <- rep(NA,N ) # number of simulation

an_b_girl <- rep(NA, N) # regression group effect

an_s_b_girl <- rep(NA, N) # error of b

an_TSS <- rep(NA, N) # Total sum of squared

an_SSREG_girl <- rep(NA, N) # Sum of square of regression for girl

an_SSE <- rep(NA, N) # sum of square error

an_MSE <- rep(NA, N) # residual variance

an_V_error <- rep(NA, N)

ac_b_y0 <- rep(NA, N) # regression coefficient for covariate (pretest)

ac_s_b_y0 <- rep(NA, N) # error of b of y0

ac_b_girl <- rep(NA, N) # regression group effect

ac_s_b_girl <- rep(NA, N) # error of b

ac_TSS <- rep(NA, N) # Total sum of squared

ac_SSREG_y0 <- rep(NA, N) # Sum of square of regression for pretest

ac_SSREG_girl <- rep(NA, N) # Sum of square of regression for girl

ac_SSE <- rep(NA, N) # sum of square error

ac_MSE <- rep(NA, N) # residual variance

ac_V_error <- rep(NA, N)

d_b_girl <- rep(NA, N) # regression group effect

d_s_b_girl <- rep(NA, N) # error of b

d_TSS <- rep(NA, N) # Total sum of squared

d_SSREG_girl <- rep(NA, N) # Sum of square of regression for girl

d_SSE <- rep(NA, N) # sum of square error

d_MSE <- rep(NA, N) # residual variance

d_V_error <- rep(NA, N)

q_b_y0 <- rep(NA, N) # regression coefficient for covariate (pretest)

q_s_b_y0 <- rep(NA, N) # error of b of y0

q_b_girl <- rep(NA, N) # regression group effect

q_s_b_girl <- rep(NA, N) # error of b

q_TSS <- rep(NA, N) # Total sum of squared

q_SSREG_y0 <- rep(NA, N) # Sum of square of regression for pretest

q_SSREG_girl <- rep(NA, N) # Sum of square of regression for girl

q_SSE <- rep(NA, N) # sum of square error

q_MSE <- rep(NA, N) # residual variance

q_V_error <- rep(NA, N)

c_b_y0 <- rep(NA, N) # regression coefficient for covariate

c_s_b_y0 <- rep(NA, N) # error of b of y0

c_b_girl <- rep(NA, N) # regression group effect

c_s_b_girl <- rep(NA, N) # error of b

c_TSS <- rep(NA, N) # Total sum of squared

c_SSREG_y0 <- rep(NA, N) # Sum of square of regression for girl

c_SSREG_girl <- rep(NA, N) # Sum of square of regression for girl

c_SSE <- rep(NA, N) # sum of square error

c_MSE <- rep(NA, N) # residual variance

c_V_error <- rep(NA, N)

for (cor in seq(-.9, 0.9, by = 0.1)) { #vary correlation between pretest and posttest

rho <- rep(cor,N)

set.seed(123456)

for (i in 1:N) {

girl <- rep(NA, NT)

y0 <- rep(NA, NT)

y1 <- rep(NA, NT)

y0_c <- rep(NA, NT)

y1_c <- rep(NA, NT)

y10 <- rep(NA, NT)

y1_dv <- rep(NA, NT)

y0_dv <- rep(NA, NT)

girl_dv <- rep(NA, NT)

# simulate the data for pretest y0 and posttest y1 for boys and girls seperately

library(mvtnorm)

varg <- matrix(c(SD^2,cov_g10 <-cor*sqrt(SD^2*SD^2),cov_g10,SD^2),ncol = 2)

varb <- matrix(c(SD^2,cov_b10 <-cor*sqrt(SD^2*SD^2),cov_b10,SD^2),ncol = 2)

g <- rmvnorm(n=NG, mean=c(M0g,M1g),sigma =varg) # generate data for girls' pretest and posttest scores

b <- rmvnorm(n=NB, mean=c(M0b,M1b),sigma =varb) #generate data for boys' pretest and posttest scores

colnames(g) <- c("y0", "y1")

colnames(b) <- c("y0", "y1")

# center the data

g <- as.data.frame(g)

b <- as.data.frame(b)

g <- g %>%

mutate (y0_c = y0 - mean(y0),

y1_c = y1 - mean(y0),

y10 = y1 - y0,

girl = 1)

b <- b %>%

mutate (y0_c = y0 - mean(y0),

y1_c = y1 - mean(y0),

y10 = y1 - y0,

girl = 0)

or.centered.data <- cbind(rbind(g,b))

or.centered.data <- or.centered.data %>%

mutate(y0_dv = y0 - mean(y0),

y1_dv = y1 - mean(y1),

girl_dv = girl - mean(girl))

library(tidyverse)

############## Testing different models

## ANOVA, using original data

ano <- lm (y1 ~ girl, data = or.centered.data)

an_b_girl[i] <- ano$coefficients[2]

an_s_b_girl[i] <- coef(summary(ano)) [2,2]

an_TSS[i] <- sum((or.centered.data$y1-mean(or.centered.data$y1))^2)

an_SSREG_girl[i] <- ano$coefficients[2] * sum((or.centered.data$girl - mean(or.centered.data$girl)) * (or.centered.data$y1 - mean(or.centered.data$y1)))

an_SSE[i] <- deviance(ano)

an_MSE[i] <- deviance(ano)/(N-1-1)

an_V_error[i] <- SD^2

## ANCOVA, using original data

ac.reg <- lm (y1 ~ y0 + girl, data = or.centered.data)

ac_b_y0[i] <- ac.reg$coefficients[2]

ac_s_b_y0[i] <- coef(summary(ac.reg)) [2,2]

ac_b_girl[i] <- ac.reg$coefficients[3]

ac_s_b_girl[i] <- coef(summary(ac.reg)) [3,2]

ac_TSS[i] <- sum((or.centered.data$y1-mean(or.centered.data$y1))^2)

ac_SSREG_y0[i] <- ac.reg$coefficients[2] * sum((or.centered.data$y0 - mean(or.centered.data$y0)) *

(or.centered.data$y1 - mean(or.centered.data$y1)))

ac_SSREG_girl[i] <- ac.reg$coefficients[3] * sum((or.centered.data$girl - mean(or.centered.data$girl)) * (or.centered.data$y1 - mean(or.centered.data$y1)))

ac_SSE[i] <- deviance(ac.reg)

ac_MSE[i] <- deviance(ac.reg)/(N-2-1)

ac_V_error[i] <- SD^2*(1-cor^2)

## difference-in-difference, using original data

DinD <- lm (y10 ~ girl, data = or.centered.data)

d_b_girl[i] <- DinD$coefficients[2]

d_s_b_girl[i] <- coef(summary(DinD)) [2,2]

d_TSS[i] <- sum((or.centered.data$y10-mean(or.centered.data$y10))^2)

d_SSREG_girl[i] <- DinD$coefficients[2] * sum((or.centered.data$girl - mean(or.centered.data$girl)) * (or.centered.data$y10 - mean(or.centered.data$y10)))

d_SSE[i] <- deviance(DinD)

d_MSE[i] <- deviance(DinD)/(N-1-1)

d_V_error[i] <- SD^2+SD^2-2*cor*(SD*SD)

## quasi-ANCOVA

q.reg <- lm (y1 ~ y0_c + girl, data = or.centered.data)

q_b_y0[i] <- q.reg$coefficients[2]

q_s_b_y0[i] <- coef(summary(q.reg)) [2,2]

q_b_girl[i] <- q.reg$coefficients[3]

q_s_b_girl[i] <- coef(summary(q.reg)) [3,2]

q_TSS[i] <- sum((or.centered.data$y1-mean(or.centered.data$y1))^2)

q_SSREG_y0[i] <- q.reg$coefficients[2] * sum((or.centered.data$y0_c - mean(or.centered.data$y0_c)) * (or.centered.data$y1 - mean(or.centered.data$y1)))

q_SSREG_girl[i] <- q.reg$coefficients[3] * sum((or.centered.data$girl - mean(or.centered.data$girl)) * (or.centered.data$y1 - mean(or.centered.data$y1)))

q_SSE[i] <- deviance(q.reg)

q_MSE[i] <- deviance(q.reg)/(N-2-1)

q_V_error[i] <- SD^2*(1-cor^2)

## Dual-centered ANCOVA: type I error

c.reg <- lm (y1_c ~ y0_c + girl, data = or.centered.data)

c_b_y0[i] <- c.reg$coefficients[2]

c_s_b_y0[i] <- coef(summary(c.reg)) [2,2]

c_b_girl[i] <- c.reg$coefficients[3]

c_s_b_girl[i] <- coef(summary(c.reg)) [3,2]

c_TSS[i] <- sum((or.centered.data$y1_c-mean(or.centered.data$y1_c))^2)

c_SSREG_y0[i] <- c.reg$coefficients[2] * sum((or.centered.data$y0_c - mean(or.centered.data$y0_c)) * (or.centered.data$y1_c - mean(or.centered.data$y1_c)))

c_SSREG_girl[i] <- c.reg$coefficients[3] * sum((or.centered.data$girl - mean(or.centered.data$girl)) * (or.centered.data$y1_c - mean(or.centered.data$y1_c)))

c_SSE[i] <- deviance(c.reg)

c_MSE[i] <- deviance(c.reg)/(N-2-1)

c_V_error[i] <- SD^2*(1-cor^2)

id[i] <- i

output <- data.frame(rho,

an_b_girl, an_s_b_girl, an_TSS, an_SSREG_girl, an_SSE, an_MSE, an_V_error,

ac_b_y0, ac_s_b_y0, ac_b_girl, ac_s_b_girl, ac_TSS, ac_SSREG_y0, ac_SSREG_girl, ac_SSE, ac_MSE, ac_V_error, d_b_girl, d_s_b_girl, d_TSS, d_SSREG_girl, d_SSE, d_MSE, d_V_error,

q_b_y0, q_s_b_y0, q_b_girl, q_s_b_girl, q_TSS, q_SSREG_y0, q_SSREG_girl, q_SSE, q_MSE, q_V_error, c_b_y0, c_s_b_y0, c_b_girl, c_s_b_girl, c_TSS, c_SSREG_y0, c_SSREG_girl, c_SSE, c_MSE, c_V_error)

output <- data.frame(output)

smry[d,] <- c(colMeans(output, dims = 1))

tsd[d,] <- c(sd(output$an_b_girl),

sd(output$ac_b_girl),

sd(output$d_b_girl),

sd(output$q_b_girl),

sd(output$c_b_girl))

}

d = d + 1

}

################ Organize and save the results

smry <- data.frame(smry)

smry <- cbind(smry, tsd)

colnames (smry)<- c("rho",

"an_b_girl", "an_s_b_girl", "an_TSS", "an_SSREG_girl", "an_SSE", "an_MSE", "an_v_error",

"ac_b_y0", "ac_s_b_y0", "ac_b_girl", "ac_s_b_girl", "ac_TSS", "ac_SSREG_y0", "ac_SSREG_girl", "ac_SSE", "ac_MSE", "ac_v_error",

"d_b_girl", "d_s_b_girl", "d_TSS", "d_SSREG_girl", "d_SSE", "d_MSE", "d_v_error",

"q_b_y0", "q_s_b_y0", "q_b_girl", "q_s_b_girl", "q_TSS", "q_SSREG_y0", "q_SSREG_girl", "q_SSE", "q_MSE", "q_v_error",

"c_b_y0", "c_s_b_y0", "c_b_girl", "c_s_b_girl", "c_TSS", "c_SSREG_y0", "c_SSREG_girl", "c_SSE", "c_MSE", "c_v_error",

"an_sd_b_girl", "ac_sd_b_girl", "d_sd_b_girl", "q_sd_b_girl", "c_sd_b_girl")

smry <- data.frame(smry)

smry

library(tidyverse)

data_an <- smry %>%

select("rho", "an_b_girl", "an_s_b_girl", "an_TSS", "an_SSREG_girl", "an_SSE", "an_MSE", "an_v_error", "an_sd_b_girl") %>%

mutate(b_y0 = "-", s_b_y0 = "-", SSREG_y0 = "-", Tmodel = "y1 ~ girl", m_id = 1, m_name = "ANOVA")

colnames (data_an)<- c("rho","b_girl", "s_b_girl", "TSS", "SSREG_girl", "SSE", "MSE", "v_error", "sd_b_girl", "b_y0", "s_b_y0", "SSREG_y0", "model", "m_id", "m_name")

data_ac <- smry %>%

select("rho", "ac_b_y0", "ac_s_b_y0", "ac_b_girl", "ac_s_b_girl", "ac_TSS", "ac_SSREG_y0", "ac_SSREG_girl", "ac_SSE", "ac_MSE", "ac_v_error", "ac_sd_b_girl") %>%

mutate(Tmodel = "y1~y0+girl", m_id = 2, m_name = "ANCOVA")

colnames (data_ac)<- c("rho","b_y0", "s_b_y0", "b_girl", "s_b_girl", "TSS", "SSREG_y0", "SSREG_girl", "SSE", "MSE", "v_error", "sd_b_girl", "model", "m_id", "m_name")

data_q <- smry %>%

select("rho", "q_b_y0", "q_s_b_y0", "q_b_girl", "q_s_b_girl", "q_TSS", "q_SSREG_y0", "q_SSREG_girl", "q_SSE", "q_MSE", "q_v_error", "q_sd_b_girl") %>%

mutate(Tmodel = "y1 ~ y0_c + girl", m_id = 3, m_name = "Quasi-ANCOVA")

colnames (data_q)<- c("rho","b_y0", "s_b_y0", "b_girl", "s_b_girl", "TSS", "SSREG_y0", "SSREG_girl", "SSE", "MSE", "v_error", "sd_b_girl", "model", "m_id", "m_name")

data_d <- smry %>%

select("rho", "d_b_girl", "d_s_b_girl", "d_TSS", "d_SSREG_girl", "d_SSE", "d_MSE", "d_v_error", "d_sd_b_girl") %>%

mutate(b_y0 = "-", s_b_y0 = "-", SSREG_y0 = "-", Tmodel = "y10 ~ girl", m_id = 4, m_name = "dif_in_dif")

colnames (data_d)<- c("rho","b_girl", "s_b_girl", "TSS","SSREG_girl", "SSE", "MSE",

"v_error", "sd_b_girl","b_y0", "s_b_y0", "SSREG_y0", "model", "m_id", "m_name")

data_c <- smry %>%

select("rho", "c_b_y0", "c_s_b_y0", "c_b_girl", "c_s_b_girl", "c_TSS", "c_SSREG_y0", "c_SSREG_girl", "c_SSE", "c_MSE", "c_v_error", "c_sd_b_girl") %>%

mutate(Tmodel = "y1_c ~ y0_c + girl", m_id = 9, m_name = "Dual-Centered")

colnames (data_c)<- c("rho","b_y0", "s_b_y0", "b_girl", "s_b_girl", "TSS", "SSREG_y0", "SSREG_girl", "SSE", "MSE", "v_error", "sd_b_girl", "model", "m_id", "m_name")

smry_prm <- cbind(rbind(data_an, data_ac, data_d, data_q, data_c))

smry_prm <- smry_prm[,c("m_id", "m_name", "model", "rho", "b_y0", "s_b_y0", "b_girl", "s_b_girl", "sd_b_girl", "TSS", "SSREG_y0", "SSREG_girl", "SSE", "MSE", "v_error")]

smry_prm

## Please change the location to save the data

setwd("G:\\...")

write.csv(smry_prm,"smry_prm.csv",row.names=FALSE)

## To match columns of Table 1 in the paper, Paper:column is Spreadsheet:B

## Paper:coumns 2-6 are spreadsheet:D-I, Paper:comun6 is spreadsheet:N

## Numbers can differ slightly due to different random number streams being used
