# Supplementary material for: Critique of enhanced power claimed for Quasi-ANCOVA and Dual-Centered ANCOVA
Source: PLoS One. 2025 Jan 22;20(1):e0317860. doi: 10.1371/journal.pone.0317860 (PMC11753683; doi:10.1371/journal.pone.0317860)
Supplement: Attachment — Submitted filename: Response To Reviewers.docx [file pone.0317860.s002.docx]

Response To Reviewers

The one request of either reviewer was to explain the subscript 1 after equation (1) and we have done so.
